# Association of socioeconomic status with hearing loss in Chinese working-aged adults: A population-based study

**DOI:** 10.1371/journal.pone.0195227

**Published:** 2018-03-29

**Authors:** Ping He, Yanan Luo, Xiangyang Hu, Rui Gong, Xu Wen, Xiaoying Zheng

**Affiliations:** 1 China Center for Health Development Studies, Peking University, Beijing, China; 2 Institute of Population Research, Peking University, Beijing, China; 3 Bloomberg School of Public Health, Johns Hopkins University, Baltimore, MD, United States of America; 4 China Rehabilitation Research Center for Deaf Children, Beijing, China; 5 APEC Health Science Academy (HeSAY), Peking University, Beijing, China; Duke University School of Medicine and Duke Global Health Institute, UNITED STATES

## Abstract

Hearing loss is the most common sensory impairment, but limited studies focused on the association of socioeconomic status (SES) with hearing loss among adults of working age. This paper aimed to fill this gap among Chinese adults. We obtained data from Ear and Hearing Disorder Survey conducted in four provinces of China in 2014–2015. The survey was based on WHO Ear and Hearing Disorders Survey Protocol and 25,860 adults aged 25 to 59 years were selected in this study. Trained local examiners performed pure tone audiometry to screen people with hearing loss, and those who were screened positively for hearing loss were referred to audiologists to make final diagnosis. SES was measured by occupation, education and income. Results show after adjusting for SES measures and covariates, in urban areas, compared with white-collar workers, blue-collar workers and the unemployed were more likely to have hearing loss, with an odds ratio of 1.2 (95%CI: 1.0, 1.3) and 1.2 (95%CI: 1.0, 1.4), respectively. Compared with people with education of senior high school or above, those with junior high school, primary school and illiteracy had 1.6 (95%CI: 1.4, 1.8), 2.1(95%CI: 1.7, 2.5) and 2.6 (95%CI: 1.9, 3.7) times as likely to have hearing loss, respectively. In rural areas, the unemployed had 1.5 (95%CI: 1.0, 2.3) times the risk of hearing loss compared with white-collar workers, and illiterates had 1.6 (95%CI: 1.6, 2.1) times the risk of hearing loss compared with people with education of senior high school or above, after SES variables and covariates were taken into considerations. Income was not significantly associated with hearing loss in urban and rural areas. In conclusion, SES, in the form of occupation and education, was associated with hearing loss among working-aged population, and further studies are needed to explore the mechanism of such association.

## Introduction

Hearing loss is the most common sensory impairment and has become a public health concern worldwide [1]. Hearing loss represents a frustrating condition, which is associated with communication difficulties, impaired cognitive functioning, and reduced quality of life [2]. The recent Global Burden of Disease Study 2013 showed that hearing loss had been ranked as the fifth leading cause of years lived with disability, higher than other chronic diseases including diabetes, dementia, and chronic obstructive pulmonary disease[1, 3]. In china, a study found that 10.41% of people aged 15–59 years were diagnosed with hearing loss in 2015[4].

Working-aged adults, defined as people aged 25–59 years in China, accounted for roughly 53% of the total population[5]. An increasing number of individuals in this age group had diabetes, hypertension and noise exposure, and the presence of these conditions were associated with higher rates of hearing loss[6, 7]. In addition, working-aged people often face multiple physical and psychological stresses, such as stressful responsibility, job dissatisfaction and mental stress at work[8, 9], and therefore these stresses interacted with complex feed-back neuroendocrine systems and immune system could contribute to hearing loss[10].

Socioeconomic status (SES), is associated with hearing loss[11–14], but studies on this topic among working-aged population are realtively limited[15]. Studies have shown that lower SES was associated with higher risk of hearing loss in adults of working age[15, 16]. Occupation, education and income were commonly used as SES measures to examine their relationship with health in China[17, 18]. Up to now, there are no studies on the association between SES and hearing loss in Chinese working-aged adults. Therefore, more knowledge about this issue is needed for this population in China.

In this study, using a cross-sectional, population-based Ear and Hearing Disorder Survey in four provinces of China, we aimed to examine the relationship between SES and hearing loss in Chinese adults aged 25 to 59 years. This study will fill the gap on this topic for China and contribute to world literature in the context of eastern countries.

## Methods

### Participants

We obtained data from Ear and Hearing Disorder Survey, which was a population-based study conducted in four provinces of China (Jilin, Guangdong, Gansu, and Shaanxi) between August 2014 and September 2015. The survey design was established by a technical team based on WHO Ear and Hearing Disorder Survey Protocol [19], which has been used in China [20–23]. The screening scale of audiometry was on the basis of the modified version of the WHO/PBD Ear and Hearing Disorders Examination Form (Version 8.3)[24].

The sampling frame covered almost 200 million people, representing about one in seven of the total population in China. Probability proportion to size (PPS) sampling method was used to identify 144 sites from 24 counties or districts in four provinces. Each site included 100 households which had lived in the registered address for over 6 months. A total of 47,511 individuals were randomly selected and 45,052 of them participated in the survey, yielding a participation rate of 94.8% [4]. All participants consented to participate in the survey, and if required, to be subsequently examined by audiologists.

**Fig 1:**
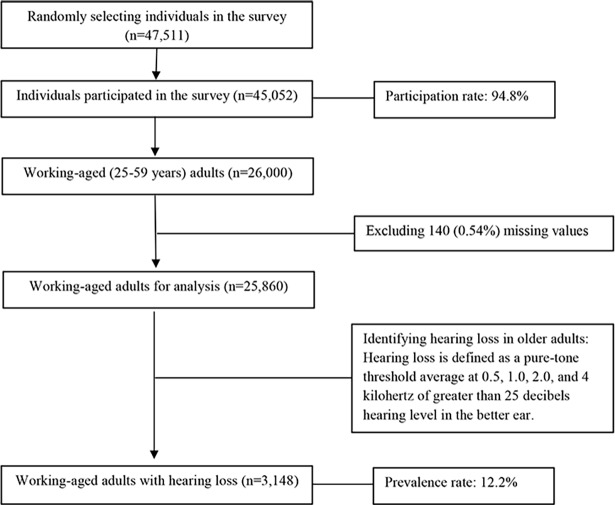
Flowchart of the study sample.

In this study, we restricted our analysis to 26,000 adults at the ages between 25 and 59 years, and after excluding 160 missing values, we obtained 25,840 individuals for analysis. Fig 1 presents more details on the sample selection of this study.

### Audiometric assessment

Audiometry was performed by trained examiners according to the established protocols of Ear and Hearing Disorder Survey. Examiners were recruited from local primary health care institutions (village doctors in rural area and community physicians in urban area), and were trained by provisional technical teams in survey skills and audiological techniques. Before the household survey, examiners went to local sites to select audiometric test rooms with ambient noise levels not exceeding 40 dBA measured by sound level meters, and were equipped with MADSEN-Xeta pure tone audiometers and other required examination equipment. During the survey, noise-excluding headsets would be used if the ambient noise of testing rooms exceeded 40dB. Audiometric equipment was calibrated by a laboratory at the beginning and end if a study, and on a daily basis by team members using self-calibration against their known hearing levels. During and after the survey, a subsample with 5% of participants were rechecked and the consistence rates of rechecks both reached 90% and above [4].

Examiners performed pure tone audiometry among people aged 25–59 years in a selected quiet room. Both ears were tested separately at 0.5, 1, 2, and 4 kHz to obtain the hearing threshold on each frequency point. There were no people who could not have both ears tested due to injury or ear infection in our survey. For those who could only have one ear tested, we performed audiometry in the better ear. Pure tone threshold averages in the better ear were calculated to identify grades of hearing loss according to the standard of WHO/PDH/97.3 [19], and the categories were collapsed into two groups: not having hearing loss (≤ 25dB) and having hearing loss (> 25dB). Afterwards, those suspected patients with hearing loss were referred to audiologists for final diagnosis of hearing loss. The referred audiological data were used in this study.

## Ethics approval

This survey was ethically approved by China Disabled Persons' Federation (number: 2014&ZZ028). The committee board, which was composed of audiologists and epidemiologists, reviewed the study protocols and ethical situations. The authors had no access to identifying information for the study participants prior to data analysis. All participants signed the informed consent with interviewers to participate in the survey and clinical diagnosis. For those with severe hearing loss who were unable to sign the consent, family members represented them to sign the consent. We obtained data from this survey, which was performed independently from this specific study.

### Measures

The outcome variable was whether or not an adult had hearing loss. The independent variable was socioeconomic status, defined by three categorical variables: occupation (white-collar worker, farming worker, blue-collar worker, others and the unemployed), education (illiteracy, primary school, junior high school and senior high school or above) and income (quintiles of annual family income per capita). White-collar workers in this study involved professional and governmental employees, and blue-collar workers referred to manual and services-oriented workers excluding farmers[25]. Covariates included gender (male and female), having spouse (yes and no) and age group (25–29, 30–39, 40–49 and 50–59 years). The independent variables and covariates were all self-reported.

### Analytical approach

Logistic regression models were used to evaluate multivariate associations between hearing loss and SES variables, and the odds ratios (ORs) with 95% confidence intervals (CIs) were presented. Although socioeconomic indicators are correlated, occupation, education and income are not interchangeable [26]. Therefore, we need to control for each socioeconomic indicator and related covariates to obtain the “net effect” of each SES measure. A P value less than 0.05 was considered statistically significant. The software Stata version 13.0 for Windows (Stata Corp, College Station, TX, USA) was utilized for the statistical analysis. All analyses were conducted separately by urban and rural areas.

## Results

Table 1 shows the characteristics of participants by urban and rural samples. In urban areas, among individuals with hearing loss, 39.2% of them were blue-collar workers, 50.9% completed education of senior high school or above, 34.7% were from the highest-income families, 52.4% aged 50–59 years, 56.5% were male, and 89.6% had a spouse. In rural areas, among individuals with hearing loss, 78.0% of them were farmers, 43.7% completed education of junior high school, 33.9% were from the lowest-income families, 53.9% aged 50–59 years, 55.0% were male, and 93.2% had a spouse.

**Table 1:** Characteristics of adults aged 25–59 years, by urban and rural area: Ear and Hearing Disorder Survey in four provinces of China, 2014–2015.

Characteristics	Urban, n (%)		Rural, n (%)	
	Not having hearing loss (n = 12,014)	Having hearing loss (n = 1,709)	Not having hearing loss (n = 10,698)	Having hearing loss (n = 1,439)
Independent Variables				
Occupation				
White collar	4428(36.9)	444(26.0)	638(6.0)	53(3.7)
Farming	439(3.7)	82(4.8)	8371(78.3)	1123(78.0)
Blue collar	4090(34.0)	670(39.2)	1228(11.5)	164(11.4)
Other	560(4.7)	83(4.9)	159(1.5)	23(1.6)
Unemployed	2497(20.8)	430(25.2)	302(2.8)	76(5.3)
Education				
Senior high school or above	8231(68.5)	870(50.9)	1497(14.0)	119(8.3)
Junior high school	2990(24.9)	599(35.1)	5910(55.2)	629(43.7)
Primary school	624(5.2)	179(10.5)	2791(26.1)	544(37.8)
Illiteracy	169(1.4)	61(3.6)	500(4.7)	147(10.2)
Income				
Quintile 1 (highest)	4933(41.1)	593(34.7)	698(6.5)	108(7.5)
Quintile 2	2348(19.5)	345(20.2)	1188(11.1)	183(12.7)
Quintile 3	2083(17.3)	327(19.1)	2454(22.9)	285(19.8)
Quintile 4	1624(13.5)	257(15.0)	2883(27.0)	375(26.1)
Quintile 5 (lowest)	1026(8.5)	187(10.9)	3475(32.5)	488(33.9)
Covariates				
Age group, years				
25–29	1459(12.1)	34(2.0)	1955(18.3)	40(2.8)
30–39	3937(32.8)	194(11.4)	2956(27.6)	130(9.0)
40–49	4182(34.8)	585(34.2)	3587(33.5)	494(34.3)
50–59	2436(20.3)	896(52.4)	2200(20.6)	775(53.9)
Gender				
Female	6330(52.7)	744(43.5)	5334(49.9)	648(45.0)
Male	5684(47.3)	965(56.5)	5364(50.1)	791(55.0)
Having spouse				
No	1495(12.4)	177(10.4)	839(7.8)	98(6.8)
Yes	10519(87.6)	1532(89.6)	9859(92.2)	1341(93.2)

Table 2 presents the prevalence of hearing loss in adults aged 25–59 years in urban and rural areas. In both urban and rural areas, white-collar workers had the lowest prevalence of hearing loss. The prevalence of hearing loss increased with decreasing level of education in both urban and rural areas. Hearing loss seemed not to be associated with income in urban areas, but to be associated with lower level of family income in rural areas. In both urban and rural areas, the prevalence of hearing loss increased with increasing age. Males and people having a spouse had higher prevalence of hearing loss in both urban and rural areas.

**Table 2:** Prevalence (%) with 95% confidential intervals of hearing loss in adults aged 25–59 years: Ear and Hearing Disorder Survey in four provinces of China, 2014–2015.

	Urban	Rural
Independent variables		
Occupation		
White collar	7.7(5.9,9.9)	9.1(8.3,10.0)
Farming	11.8(11.2,12.5)	15.7(12.9,19.1)
Blue collar	11.8(10.2,13.6)	14.1(13.1,15.1)
Other	12.6(8.5,18.3)	12.9(10.5,15.7)
Unemployed	20.1(16.4,24.5)	14.7(13.5,16.0)
Education		
Senior high school or above	7.4(6.2,8.7)	9.6(9,10.2)
Junior high school	9.6(8.9,10.4)	16.7(15.5,17.9)
Primary school	16.3(15.1,17.6)	22.3(19.5,25.3)
Illiteracy	22.7(19.7,26.1)	26.5(21.2,32.6)
Income		
Quintile 1 (highest)	13.4(11.2,15.9)	10.7(9.9,11.6)
Quintile 2	13.3(11.6,15.3)	12.8(11.6,14.1)
Quintile 3	10.4(9.3,11.6)	13.6(12.3,15)
Quintile 4	11.5(10.5,12.7)	13.7(12.2,15.3)
Quintile 5 (lowest)	12.3(11.3,13.4)	15.4(13.5,17.6)
Covariates		
Age group, years		
25–29	2.0(1.5,2.7)	2.3(1.6,3.2)
30–39	4.2(3.6,5)	4.7(4.1,5.4)
40–49	12.1(11.1,13.1)	12.3(11.4,13.2)
50–59	26.1(24.5,27.7)	26.9(25.4,28.4)
Gender		
Female	10.8(10.1,11.6)	10.5(9.8,11.3)
Male	12.9(12,13.7)	14.5(13.7,15.4)
Having spouse		
No	10.5(8.7,12.6)	10.6(9.2,12.2)
Yes	12.0(11.4,12.6)	12.7(12.1,13.3)

Table 3 shows logistic regressions on the association between SES and hearing loss among adults aged 25–59 years in urban areas. In Model 1 containing all SES measures, income was not significantly associated with hearing loss, but occupation and education were significantly associated with hearing loss. After further adjusting for gender, having spouse and age group in Model 2 and Model 3, the association of occupation and education with hearing loss remained significant despite its magnitude became smaller. In Model 3, compared with white-collar workers, blue-collar workers and the unemployed were more likely to have hearing loss, with an odds ratio of 1.2 (95%CI: 1.0, 1.3) and 1.2 (95%CI: 1.0, 1.4), respectively. Compared with people with education of senior high school or above, those with junior high school, primary school and illiteracy had 1.6 (95%CI: 1.4, 1.8), 2.1(95%CI: 1.7, 2.5) and 2.6 (95%CI: 1.9, 3.7) times as likely to have hearing loss, respectively.

**Table 3:** Odds ratios with 95% confidential intervals in the association between socioeconomic status and hearing loss in adults aged 25–59 years in urban areas.

	Model 1	Model 2	Model 3
Independent variables			
Occupation			
White collar	Reference	Reference	Reference
Farming	1.1(0.9,1.5)	1.1(0.8,1.5)	0.9(0.6,1.1)
Blue collar	1.4(1.2,1.6) ***	1.3(1.2,1.5) ***	1.2(1.0,1.3) *
Other	1.1(0.9,1.5)	1.1(0.9,1.5)	1.0(0.8,1.4)
Unemployed	1.2(1.0,1.4) *	1.3(1.1,1.5) **	1.2(1.0,1.4) *
Education			
Senior high school or above	Reference	Reference	Reference
Junior high school	1.8(1.6,2.0) ***	1.8(1.6,2.0) ***	1.6(1.4,1.8) ***
Primary school	2.6(2.1,3.1) ***	2.7(2.2,3.3) ***	2.1(1.7,2.5) ***
Illiteracy	3.3(2.4,4.5) ***	3.6(2.6,4.9) ***	2.6(1.9,3.7) ***
Income			
Quintile 1 (highest)	Reference	Reference	Reference
Quintile 2	1.1(0.9,1.3)	1.1(0.9,1.3)	1.1(0.9,1.3)
Quintile 3	1.0(0.9,1.2)	1.0(0.9,1.2)	1.0(0.9,1.2)
Quintile 4	1.0(0.8,1.1)	1.0(0.8,1.1)	0.9(0.8,1.1)
Quintile 5 (lowest)	1.0(0.9,1.2)	1.0(0.8,1.2)	1.0(0.8,1.3)
Covariates			
Gender			
Female		Reference	Reference
Male		1.6(1.4,1.7) ***	1.5(1.4,1.7) ***
Having spouse			
No		Reference	Reference
Yes		1.2(1.0,1.5) *	0.9(0.7,1.0)
Age group, years			
25–29			Reference
30–39			2.1(1.5,3.1) ***
40–49			5.5(3.9,7.9) ***
50–59			14.3(10.0,20.5) ***

**Note:** Model 1 adjusted for occupation, education, and income; Model 2 adjusted for occupation, education, income, gender, and marital status; Model 3 adjusted for occupation, education, income, gender, marital status, and age group.

*P<0.05

**P <0.01

***P <0.001.

Table 4 presents logistic regressions on the association between SES and hearing loss among adults aged 25–59 years in rural areas. In Model 1 and Model 2, occupation, education and income were each significantly associated with hearing loss. In Model 3 which further adjusted for age group, the association of income with hearing loss became not significant, and the association of occupation and education with hearing loss remained significant and its magnitude turned out to be weaker. The unemployed had 1.5 (95%CI: 1.0, 2.3) times the risk of hearing loss compared with white-collar workers. Illiterates had 1.6 (95%CI: 1.6, 2.1) times the risk of hearing loss compared with people with education of senior high school or above.

**Table 4:** Odds ratios with 95% confidential intervals in the association between socioeconomic status and hearing loss in adults aged 25–59 years in rural areas.

	Model 1	Model 2	Model 3
Independent variables			
Occupation			
White collar	Reference	Reference	Reference
Farming	1.2(0.9,1.6)	1.2(0.9,1.6)	0.8(0.6,1.1)
Blue collar	1.4(1,1.9)	1.4(1.0,1.9)	1.3(0.9,1.9)
Other	1.3(0.8,2.3)	1.4(0.8,2.4)	1.1(0.6,1.8)
Unemployed	2.0(1.4,3.0) ***	2.2(1.5,3.3) ***	1.5(1.0,2.3)*
Education			
Senior high school or above	Reference	Reference	Reference
Junior high school	1.4(1.1,1.7) **	1.3(1.1,1.6) **	1.0(0.8,1.3)
Primary school	2.5(2.0,3.1) ***	2.5(2.0,3.1) ***	1.2(1.0,1.5)
Illiteracy	3.8(2.9,5.0) ***	4.1(3.1,5.5) ***	1.6(1.2,2.1) **
Income			
Quintile 1 (highest)	Reference	Reference	Reference
Quintile 2	1.0(0.8,1.3)	1.0(0.8,1.3)	1.1(0.9,1.5)
Quintile 3	0.7(0.6,0.9) **	0.7(0.6,0.9) *	0.9(0.7,1.1)
Quintile 4	0.8(0.6,1.0)	0.8(0.6,1.0)	1.1(0.8,1.4)
Quintile 5 (lowest)	0.8(0.6,1.0) *	0.8(0.6,1.0) *	1.1(0.9,1.4)
Covariates			
Gender			
Female		Reference	Reference
Male		1.4(1.3,1.6) ***	1.3(1.2,1.5) ***
Having spouse			
No		Reference	Reference
Yes		1.2(1.0,1.5)	0.7(0.6,0.9) **
Age group, years			
20–29			Reference
30–39			2.4(1.6,3.4) ***
40–49			7.5(5.4,10.6) ***
50–59			19.0(13.5,26.8) ***

**Note:** Model 1 adjusted for occupation, education, and income; Model 2 adjusted for occupation, education, income, gender, and marital status; Model 3 adjusted for occupation, education, income, gender, marital status, and age group.

*P<0.05

**P <0.01

***P <0.001.

## Discussion

In this study, SES, as defined by occupation, education and income, was investigated regarding its association with hearing loss in working-aged adults in a population-based Earing and Hearing Disorder Survey in four provinces of China. According to the WHO Ear and Hearing Disorders Survey Protocol, individuals were first screened by local trained examiners using pure tone audiometry and were further referred to audiologists for final diagnosis of hearing loss. To the best of our knowledge, this is the first to investigate the association between SES and hearing loss in adults of working age from a population-based survey in China. We found that lower SES, in the form of occupation and education, was associated with higher risk of hearing loss in both urban and rural areas.

Our findings showed that lower-class occupation was correlated with higher level of hearing loss, which is consistent with previous research[27]. In this study, compared with white-collar workers, blue-collar workers and the unemployed were more likely to have hearing loss in urban areas, and the unemployed were more prone to having hearing loss in rural areas. The correlates of occupation with hearing loss were likely because of the connection with job-related exposure and social standing [28]. For instance, blue-collar workers, including manual and services workers, were more inclined to exposure to risk factors of hearing loss [29], such as ototoxic chemicals[30], working environment noise and whole-body vibration [31]. In addition, the association of the unemployed with greater risk of hearing loss is partly due to their poor hearing knowledge as well as less access to care for hearing conditions[28].

This study indicated that lower education was associated with elevated level of hearing loss when a wide range of covariates were taken into consideration, which is consistent with prior studies [11, 14]. Although little is known about the causal mechanism between education and hearing loss, we can speculate some potential reasons. For example, lower education is a marker of unhealthy lifestyle attributes, including higher prevalence of alcohol intake, smoking and obesity [27, 32], which are related to greater risk of hearing loss [12]. In addition, adults with higher level of education may have better birth hearing status [33], better access to health care for hearing-related conditions, and less workplace stress [27]. Furthermore, individuals with hearing loss have been shown to perform worse in school and are more likely to drop out early[34].

Our study found that income was not associated with hearing loss among working-aged adults in urban and rural areas. Lower income is usually linked with poor access to, utilization of, and quality of health care and then correlated with poorer health status [35]. Such mechanism may adapt to hearing conditions, leading to greater risk of hearing loss in lower-income adults. However, we did not observe the inverse association of income with hearing loss after adjusting for SES measures and covariates in urban and rural areas. This is not consistent with previous studies on the association between income and hearing impairment among working-aged adults aged 25–64 years in the United States [15], or not consistent with studies in adults aged 18–64 years in the Netherlands [36] and adults aged 20–74 years in Canada [37].

This study has several limitations. Since some factors that may affect response variability were not considered, we need to take caution to interpret our results. For instance, the main purpose of this study was to investigate the association between SES and hearing loss, but some risk factors, such as noise exposure, chronic diseases (i.e. hypertension and diabetes) and cigarette smoking, may contribute to the observed association [38]. In addition, although SES was commonly measured by occupation, education and income, research has found that failure to include wealth indicator could underestimate the effect of health [39, 40]. Furthermore, one person’s SES may be correlated with the SES of their parents and spouse. However, due to restriction of our study design, we did not include these covariates in this study. Finally, a cross-section design for this study cannot draw causal inferences. Hearing loss in early life may also result in lower levels of education, income and employment opportunities in adulthood [41]. From this perspective, further studies are necessary to investigate the causality and its pathways between SES and hearing loss drawing from prospective cohort design.

Despite these limitations, the strengths of this study include a large-size, population-based design in four provinces of China based on the WHO Ear and Hearing Disorder Survey Protocol, access to a variety of socioeconomic measures and multiple covariates in the association of SES with hearing loss, as well as hearing conditions ascertained by audiologists according to the WHO criteria.

## Conclusions

This study investigated the association between SES and hearing loss among working-aged adults aged 25 to 59 years in four provinces of China. We found that SES, in the form of occupation and education, was associated with hearing loss. However, income was shown not to be associated with hearing loss in our findings. This study contributes to the literature on hearing loss in developing nations of a non-Western context. Further studies are warranted to confirm our findings and to better understand how SES is related to hearing loss, which will help identify how to prevent hearing loss and to provide more specific suggestions to improve hearing status for working-aged population.

## References

[1] Looi LM, Ganten D, McGrath PF, Gross M, Griffin GE. Hearing loss: a global health issue. Lancet. 2015;385(9972):943–4. Epub 2015/03/07. doi: 10.1016/s0140-6736(15)60208-2 . 25743174 10.1016/S0140-6736(15)60208-2

[2] Dalton DS, Cruickshanks KJ, Klein BE, Klein R, Wiley TL, Nondahl DM. The impact of hearing loss on quality of life in older adults. The Gerontologist. 2003;43(5):661–8. 14570962 10.1093/geront/43.5.661

[3] Global, regional, and national incidence, prevalence, and years lived with disability for 301 acute and chronic diseases and injuries in 188 countries, 1990–2013: a systematic analysis for the Global Burden of Disease Study 2013. Lancet. 2015;386(9995):743–800. Epub 2015/06/13. doi: 10.1016/S0140-6736(15)60692-4 ; PubMed Central PMCID: PMCPMC4561509. 26063472 10.1016/S0140-6736(15)60692-4 PMC4561509

[4] Hu XY, Zheng XY, Ma FR, Long M, Han R, Zhou LJ, et al [Prevalence of hearing disorders in China: a population-based survey in four provinces of China]. Zhonghua Er Bi Yan Hou Tou Jing Wai Ke Za Zhi. 2016;51(11):819–25. doi: 10.3760/cma.j.issn.1673-0860.2016.11.004 . 27938607 10.3760/cma.j.issn.1673-0860.2016.11.004

[5] National Bureau of Statistics of China. 2010 Population Census. 2010 [cited 2017 September 22]. Available from: http://www.stats.gov.cn/english/Statisticaldata/CensusData/rkpc2010/indexch.htm.

[6] Hong JW, Jeon JH, Ku CR, Noh JH, Yoo HJ, Kim DJ. The prevalence and factors associated with hearing impairment in the Korean adults: the 2010–2012 Korea National Health and Nutrition Examination Survey (observational study). Medicine. 2015;94(10):e611 Epub 2015/03/12. doi: 10.1097/MD.0000000000000611 ; PubMed Central PMCID: PMCPMC4602472. 25761183 10.1097/MD.0000000000000611 PMC4602472

[7] Engdahl B, Tambs K. Occupation and the risk of hearing impairment—results from the Nord-Trondelag study on hearing loss. Scandinavian journal of work, environment & health. 2010;36(3):250–7. Epub 2009/12/22. . 20024522 10.5271/sjweh.2887

[8] Andersson GB. Epidemiological features of chronic low-back pain. The lancet. 1999;354(9178):581–5. 10.1016/S0140-6736(99)01312-4 10470716

[9] Uehata T. Long working hours and occupational stress-related cardiovascular attacks among middle-aged workers in Japan. Journal of human ergology. 1991;20(2):147–53. 1842961

[10] Horner K. The emotional ear in stress. Neuroscience & Biobehavioral Reviews. 2003;27(5):437–46. 14505685 10.1016/s0149-7634(03)00071-x

[11] Helvik AS, Krokstad S, Tambs K. Socioeconomic inequalities in hearing loss in a healthy population sample: The HUNT Study. American Journal of Public Health. 2009;99(8):1376–8. Epub 2009/06/23. doi: 10.2105/AJPH.2007.133215 ; PubMed Central PMCID: PMCPMC2707479. 19542048 10.2105/AJPH.2007.133215 PMC2707479

[12] Lee JS, Choi HG, Jang JH, Sim S, Hong SK, Lee HJ, et al Analysis of Predisposing Factors for Hearing Loss in Adults. Journal of Korean medical science. 2015;30(8):1175–82. Epub 2015/08/05. doi: 10.3346/jkms.2015.30.8.1175 ; PubMed Central PMCID: PMCPMC4520950. 26240497 10.3346/jkms.2015.30.8.1175 PMC4520950

[13] Wilson DH, Walsh PG, Sanchez L, Davis AC, Taylor AW, Tucker G, et al The epidemiology of hearing impairment in an Australian adult population. International journal of epidemiology. 1999;28(2):247–52. Epub 1999/05/26. . 10342686 10.1093/ije/28.2.247

[14] Cruickshanks KJ, Dhar S, Dinces E, Fifer RC, Gonzalez F 2nd, Heiss G, et al Hearing Impairment Prevalence and Associated Risk Factors in the Hispanic Community Health Study/Study of Latinos. JAMA otolaryngology—head & neck surgery. 2015;141(7):641–8. Epub 2015/05/30. doi: 10.1001/jamaoto.2015.0889 ; PubMed Central PMCID: PMCPMC4506256. 26021283 10.1001/jamaoto.2015.0889 PMC4506256

[15] Chou CF, Beckles GL, Zhang X, Saaddine JB. Association of Socioeconomic Position With Sensory Impairment Among US Working-Aged Adults. American Journal of Public Health. 2015;105(6):1262–8. Epub 2015/04/17. doi: 10.2105/AJPH.2014.302475 ; PubMed Central PMCID: PMCPMC4431072. 25880957 10.2105/AJPH.2014.302475 PMC4431072

[16] Hasson D, Theorell T, Westerlund H, Canlon B. Prevalence and characteristics of hearing problems in a working and non-working Swedish population. Journal of Epidemiology & Community Health. 2010;64(5):453–60. 19692714 10.1136/jech.2009.095430

[17] Guo H, Sa Z. Socioeconomic Differentials in Smoking Duration among Adult Male Smokers in China: Result from the 2006 China Health and Nutrition Survey. PloS one. 2015;10(1):e0117354 doi: 10.1371/journal.pone.0117354 25575097 10.1371/journal.pone.0117354 PMC4289072

[18] Chen M, Wu Y, Narimatsu H, Li X, Wang C, Luo J, et al Socioeconomic Status and Physical Activity in Chinese Adults: A Report from a Community-Based Survey in Jiaxing, China. PloS one. 2015;10(7):e0132918 doi: 10.1371/journal.pone.0132918 26177205 10.1371/journal.pone.0132918 PMC4503452

[19] Organization WH. WHO ear and hearing disorders survey protocol for a population-based survey of prevalence and causes of deafness and hearing impairment and other ear disorders World Health Organization 1999.

[20] Bu X. The Report of Results from the Pilot Study on WHO Ear and Hearing Disorders Survey Protocol in Jiangsu Province, China. Informal Consultation on Epidemiology of Deafness and Hearing Impairment in Developing Countries and Update of the Who Protocol WHO, Geneva. 2003.

[21] Bu X, Li X, Driscoll C. The Chinese hearing questionnaire for school children. Journal of the American Academy of Audiology. 2005;16(9):687–97. 16515140 10.3766/jaaa.16.9.6

[22] Bu X, Liu C, Xing G, Zhou L, Liang C, Zheng Y, et al WHO Ear and Hearing Disorders Survey in four provinces in China. Audiological Medicine. 2011;9(4):141–6.

[23] Smith A, Mathers C, Newton V, Vallely P. Epidemiology of infection as a cause of hearing loss. Infection and hearing impairment. 2006:31–66.

[24] World Health Organization. WHO/PBD Ear and Hearing Disorders Examination Form Version 8.3. Retrived in June, 2017, from http://www.who.int/blindness/Ear_hearingsurveyformupdtaed.pdf. 2012 Contract No.: 1 March.

[25] Wang H, Ma L, Yin Q, Zhang X, Zhang C. Prevalence of alcoholic liver disease and its association with socioeconomic status in north-eastern China. Alcoholism, clinical and experimental research. 2014;38(4):1035–41. Epub 2014/01/17. doi: 10.1111/acer.12321 . 24428769 10.1111/acer.12321

[26] Geyer S, Hemström Ö, Peter R, Vågerö D. Education, income, and occupational class cannot be used interchangeably in social epidemiology. Empirical evidence against a common practice. Journal of Epidemiology & Community Health. 2006;60(9):804–10. 16905727 10.1136/jech.2005.041319 PMC2566032

[27] Cruickshanks KJ, Nondahl DM, Tweed TS, Wiley TL, Klein BE, Klein R, et al Education, occupation, noise exposure history and the 10-yr cumulative incidence of hearing impairment in older adults. Hearing research. 2010;264(1):3–9. 19853647 10.1016/j.heares.2009.10.008 PMC2868082

[28] Galobardes B, Lynch J, Smith GD. Measuring socioeconomic position in health research. British medical bulletin. 2007;81–82:21–37. Epub 2007/02/08. doi: 10.1093/bmb/ldm001 . 17284541 10.1093/bmb/ldm001

[29] Agrawal Y, Platz EA, Niparko JK. Risk factors for hearing loss in US adults: data from the National Health and Nutrition Examination Survey, 1999 to 2002. Otology & neurotology: official publication of the American Otological Society, American Neurotology Society [and] European Academy of Otology and Neurotology. 2009;30(2):139–45. Epub 2008/12/19. doi: 10.1097/MAO.0b013e318192483c . 19092714 10.1097/MAO.0b013e318192483c

[30] Vyskocil A, Truchon G, Leroux T, Lemay F, Gendron M, Gagnon F, et al A weight of evidence approach for the assessment of the ototoxic potential of industrial chemicals. Toxicology and industrial health. 2012;28(9):796–819. Epub 2011/11/09. doi: 10.1177/0748233711425067 . 22064681 10.1177/0748233711425067

[31] Tak S, Davis RR, Calvert GM. Exposure to hazardous workplace noise and use of hearing protection devices among US workers—NHANES, 1999–2004. American journal of industrial medicine. 2009;52(5):358–71. Epub 2009/03/10. doi: 10.1002/ajim.20690 . 19267354 10.1002/ajim.20690

[32] Power C, Atherton K, Manor O. Co-occurrence of risk factors for cardiovascular disease by social class: 1958 British birth cohort. Journal of epidemiology and community health. 2008;62(12):1030–5. Epub 2008/11/15. doi: 10.1136/jech.2007.068817 . 19008367 10.1136/jech.2007.068817

[33] Zhan W, Cruickshanks KJ, Klein BE, Klein R, Huang G-H, Pankow JS, et al Modifiable determinants of hearing impairment in adults. Preventive medicine. 2011;53(4):338–42. 21871479 10.1016/j.ypmed.2011.08.012 PMC3208793

[34] Järvelin MR, Mäki–torkko E, Sorri MJ, Rantakallio PT. Effect of hearing impairment on educational outcomes and employment up to the age of 25 years in northern Finland. British journal of audiology. 1997;31(3):165–75. 9276099 10.3109/03005364000000019

[35] Adler NE, Newman K. Socioeconomic disparities in health: pathways and policies. Health affairs (Project Hope). 2002;21(2):60–76. Epub 2002/03/20. doi: 10.1377/hlthaff.21.2.60 . 11900187 10.1377/hlthaff.21.2.60

[36] Stam M, Kostense P, Festen J, Kramer S. The relationship between hearing status and the participation in different categories of work: demographics. Work. 2013;46(2):207–19. doi: 10.3233/WOR-131747 24004810 10.3233/WOR-131747

[37] Feder K, Michaud D, Ramage-Morin P, McNamee J, Beauregard Y. Prevalence of hearing loss among Canadians aged 20 to 79: Audiometric results from the 2012/2013 Canadian Health Measures Survey. Health reports. 2015;26(7):18–25. Epub 2015/07/16. . 26177043

[38] Cheng YJ, Gregg EW, Saaddine JB, Imperatore G, Zhang X, Albright AL. Three decade change in the prevalence of hearing impairment and its association with diabetes in the United States. Preventive medicine. 2009;49(5):360–4. doi: 10.1016/j.ypmed.2009.07.021 19664652 10.1016/j.ypmed.2009.07.021

[39] Pollack CE, Chideya S, Cubbin C, Williams B, Dekker M, Braveman P. Should health studies measure wealth?: A systematic review. American journal of preventive medicine. 2007;33(3):250–64. doi: 10.1016/j.amepre.2007.04.033 17826585 10.1016/j.amepre.2007.04.033

[40] Cubbin C, Pollack C, Flaherty B, Hayward M, Sania A, Vallone D, et al Assessing alternative measures of wealth in health research. American Journal of Public Health. 2011;101(5):939–47. doi: 10.2105/AJPH.2010.194175 21252050 10.2105/AJPH.2010.194175 PMC3076388

[41] Emmett SD, Francis HW. The Socioeconomic Impact of Hearing Loss in US Adults. Otol Neurotol. 2015;36(3):545–50. PubMed PMID: WOS:000349769700035. doi: 10.1097/MAO.0000000000000562 25158616 10.1097/MAO.0000000000000562 PMC4466103

